# Effects of Different Drying Methods on the Active Ingredients of Ginger Powder

**DOI:** 10.3390/foods15173144

**Published:** 2026-09-04

**Authors:** Kehong Li, Houjun Ma, Juncheng Wu, Junhong Ge, Jiale Liu, Wanlong Jiang, Hang Zou, Yafen Zhang

**Affiliations:** 1 Key Laboratory of Microbiological Metrology, Measurement & Bio-Product Quality Security, State Administration for Market Regulation, College of Life Sciences, China Jiliang University, Hangzhou 310018, China; kehongli610@163.com (K.L.); mahoujunm@126.com (H.M.); liujiale121012@163.com (J.L.); 2 College of Modern Science and Technology, China Jiliang University, Yiwu 322002, China; wujun461@gmail.com (J.W.); junhong_ge@163.com (J.G.); wljiang0112@163.com (W.J.)

**Keywords:** ginger powder, two-stage drying, gingerol, antioxidant activity, quality control

## Abstract

To evaluate the active ingredients of ginger powder processed by two-stage drying (120 °C, 90 min + 80 °C, 150 min) versus conventional drying (80 °C, 360 min), three ginger varieties—Xiaolin Huangjiang, Rizhao Shengjiang, and Luoping Xiaohuangjiang—were systematically compared. Key physicochemical indices including moisture, ash, protein, crude fiber, volatile oil, and gingerols (6-, 8-, 10-gingerol, and 6-shogaol) were determined. The results revealed that 120–80 °C two-stage drying significantly enhanced gingerol accumulation from 7.12 mg/g at 80 °C constant drying to 11.87 mg/g at 120–80 °C two-stage drying and antioxidant capacity (DPPH, ABTS, FRAP) of Xiaolin Huangjiang powder, while 80 °C conventional constant drying better retained volatile oil with a content of 3.46% compared to 1.29% under staged drying. Systematic homogeneity and stability verification confirmed that the developed Xiaolin Huangjiang QC samples exhibited excellent uniformity and stability, with definite characteristic component values and quantified uncertainty. This work provides a reliable standard and technical support for standardized processing and high-value utilization of high-quality dried ginger products.

## 1. Introduction

Ginger (*Zingiber officinale* Roscoe), a perennial herb belonging to the Zingiberaceae family, has long been widely utilized as a spice, condiment, and traditional medicine. Due to its distinct flavor profile and rich composition of bioactive constituents, ginger has garnered significant attention in both the food industry and scientific research [1]. As a primary processed derivative, ginger powder not only retains a portion of the nutritional and functional characteristics of fresh ginger but also offers advantages such as storage stability, ease of transport, and versatile application scenarios, making it highly favored by both consumers and food manufacturers. The nutritional and functional quality of ginger powder is the core determinant of its market value and application prospects. Existing literature indicates that ginger is abundant in bioactive substances, including gingerols, volatile oils, and phenolics [2]. Among these, gingerols are the characteristic components responsible for the pungent flavor and possess various physiological activities, such as antioxidant, anti-inflammatory, and antimicrobial properties [3,4]. Furthermore, nutritional components such as proteins, dietary fibers, and minerals contribute to the high nutritional value of ginger. Research has confirmed that the retention rates of these components are closely correlated with processing techniques [5,6,7]. However, the transformation of fresh ginger into powder involves multiple unit operations, each of which potentially influences the retention of bioactive and nutritional compounds, thereby affecting the final product quality. Currently, systematic studies regarding the comprehensive impact of different processing stages on the core quality indicators of ginger powder remain fragmented. Drying constitutes a critical unit operation in ginger powder production, directly influencing moisture content, shelf-life, and the retention of bioactive ingredients. Traditional drying protocols typically employ a single-temperature, long-duration mode, which may induce excessive degradation of thermosensitive bioactive compounds (e.g., gingerols and volatile oils), consequently impairing the functional quality of the powder [6]. In recent years, drying technologies, such as multi-stage temperature-controlled drying, have been progressively applied in agricultural product processing to balance drying efficiency with quality retention [8]. While studies have demonstrated the efficacy of these methods in spices like garlic, chili peppers, and jujubes [9,10,11], their application in ginger drying remains in the preliminary stages. Moreover, there is a lack of systematic evaluation regarding the effects of different drying protocols on the quality of ginger powder across different varieties. For instance, Chen et al. [12] investigated the effect of a single drying method on gingerol content in a single ginger cultivar but did not address the comprehensive comparison of multiple varieties and drying techniques. Therefore, there is an urgent need to explore drying protocols suitable for the production of high-quality ginger powder.

Beyond processing technology, the genetic background of the ginger variety is another pivotal factor influencing powder quality. Different ginger cultivars often exhibit significant variations in bioactive and nutritional composition due to differences in growth environments, genetic characteristics, and metabolic pathways [13]. Xiaolin Huangjiang, Rizhao Shengjiang, and Luoping Xiao Huangjiang are three widely cultivated ginger varieties in different regions of China, each possessing unique quality characteristics. While previous studies have compared the quality of fresh rhizomes among certain varieties [14], there is a paucity of comparative research on the physicochemical properties, bioactive composition, and antioxidant capacity of these varieties following different drying treatments. This knowledge gap hinders the screening of superior cultivars and the optimization of processing techniques for high-value ginger products.

Quality control (QC) is paramount for the standardized production of ginger products. Selecting appropriate core markers for the preparation and evaluation of QC samples is essential for ensuring product homogeneity, stability, and reproducibility [5]. As the characteristic active components of ginger, gingerols hold great potential as core markers for quality control. Although Zhong et al. [15] preliminarily confirmed the stability of gingerols in ginger powder of a single variety, further verification is required regarding the stability of gingerols across different varieties under drying and storage conditions, as well as the reliability of QC analysis. This remains a major bottleneck in the current standardized production of ginger products.

In light of the aforementioned research gaps, this study uses three ginger varieties to systematically evaluate the effects of drying protocols on the varietal differences physicochemical properties, bioactive components, antioxidant capacity, and quality stability of ginger powder. Two drying methods were employed: an two-stage drying process (high-temperature rapid drying at 120 °C for 90 min followed by low-temperature thorough drying at 80 °C for 150 min) and a conventional long-duration drying method (80 °C for 360 min). Quantitative analyses were conducted on key quality indicators, including moisture, ash, volatile oil, protein, crude fiber, and gingerol content. Additionally, antioxidant activities were assessed using DPPH, ABTS, and FRAP assays, with gingerols as the core marker for QC sample preparation and evaluation. The objective of this study is to elucidate the key factors affecting ginger powder quality, screen for superior ginger varieties, and drying processes, thereby providing a theoretical basis and technical support for the production of high-value functional ginger products and the establishment of standardized quality control systems.

## 2. Materials and Methods

### 2.1. Reagents and Instruments

Ginger: Xiaolin Huangjiang, Rizhao Shengjiang, and Luoping Xiao huangjiang, (all products were purchased from the Hangzhou Agricultural and Sideline Products Logistics Center (120.1061° E, 30.3770° N), located in Hangzhou, Zhejiang Province, China); 6-Gingerol Standard Reference Material, 8-Gingerol Standard Reference Material, 10-Gingerol Standard Reference Material, 6-Shogaol Standard Reference Material [Purchased from YR Bio (YIRUI Biotech), purity > 98%, Cheng Du, China].

UV-Vis spectrophotometer (UV-1600B, Shanghai Meipuda Instrument Co., Ltd., Shanghai, China), high-performance liquid chromatography (HPLC) system (Waters E2695, Waters Corporation, Milford, MA, USA), Pulverizing blender (Yongkang Sufeng Industry & Trade Co., Ltd., Jinhua, Yongkang, China), and GP-C18 analytical column (250 × 4.5 mm, 5 μm).

### 2.2. Preparation of Ginger Powder

**Figure 1:**
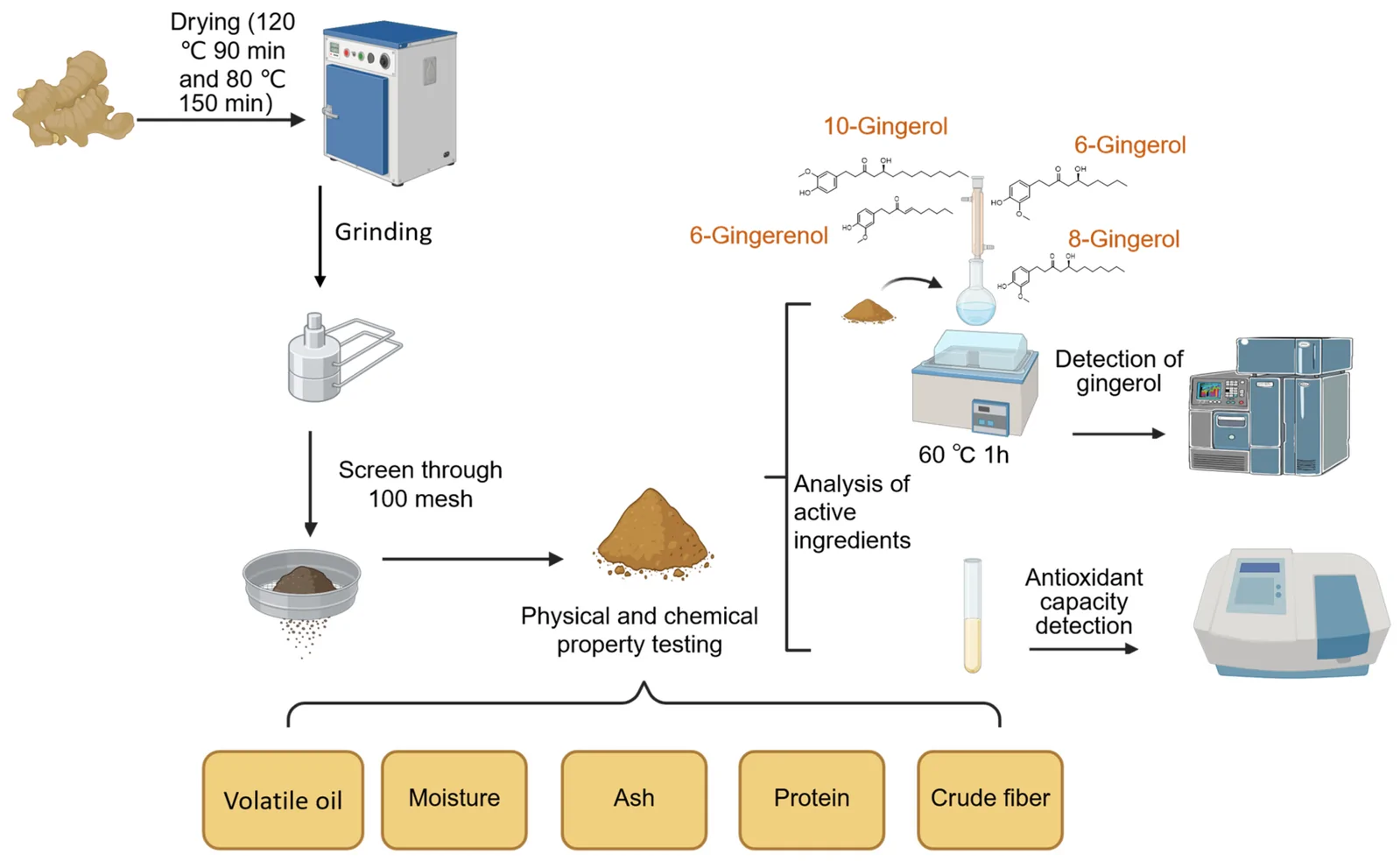
Two-stage drying: 120 °C Rapid Drying (90 min) + 80 °C Complete Drying (150 min).

Group A two-stage drying: Rapid drying at 120 °C for 90 min, followed by thorough drying at 80 °C for 150 min (Figure 1).

Group B conventional drying: Continuous drying at 80 °C for 360 min.

Fresh ginger rhizomes, with an initial moisture content of 90% (wet basis), were thoroughly washed and cut into thin strips with a thickness of 4 mm. For each batch, 400 g of fresh ginger was distributed in a single layer on the drying tray. Drying was performed in a forced-air drying oven at an air velocity of 1.0 m·s^−1^. The staged regimen consisted of drying at 120 °C for 90 min followed by 80 °C for 150 min, whereas the constant-temperature regimen consisted of drying at 80 °C for 360 min. Drying was terminated upon completion of the predetermined temperature–time schedule. The samples were then cooled to room temperature, ground into a fine powder, sieved through a 100-mesh sieve, and stored in a light-resistant environment to prevent oxidative deterioration until subsequent analysis.

Each treatment was performed with three independent biological replicates; each biological replicate was further measured in triplicate as technical replicates during chemical analysis. After drying, the samples were ground into a fine powder, sieved through a 100-mesh sieve to ensure uniformity, and then stored in a light-resistant environment to prevent oxidative deterioration.

### 2.3. Determination of Moisture Content

A 5 g sample of ginger powder was weighed and placed in an aluminum crucible. The sample was then heated in a drying oven at 105 °C for 5 h. After drying, the crucible was removed, cooled to room temperature in a desiccator, and reweighed. The moisture content percentage was calculated using Equation (1):

(1) $${\mathrm{MC}}_{\mathrm{wb}}=\frac{\left(m_{1}-m_{2}\right)}{m_{1}}\times 100\%$$

MC_wb_ is the moisture content of the sample on a wet basis (%), m_1_ is the initial weight of the sample (g), m_2_ is the weight of the sample after drying (g).

### 2.4. Determination of Ash Content

A 5 g sample of ginger powder was weighed into a ceramic crucible. The crucible was then placed in a muffle furnace and heated to approximately 550 °C, maintaining this temperature for about 2 h or until white ash was obtained. After ashing, the furnace was turned off and allowed to cool before opening. The crucible was then promptly transferred to a desiccator containing a porcelain plate and desiccant to cool to room temperature. Once cooled, the crucible was reweighed. The ash content percentage was calculated using Equation (2):

(2) $$\mathrm{AC}=\frac{W_{o}-W_{c}}{W_{f}-W_{c}}\times 100\%$$

AC is the ash content of the sample (%). W_f_ is the initial weight of the sample plus crucible (g), W_o_ is the final weight of the sample plus crucible after ashing (g), W_c_ is the weight of the empty crucible (g).

### 2.5. Determination of Protein Content

The protein content of ginger powder was determined using the Kjeldahl method. A 1.5 g sample was weighed into a dry Kjeldahl digestion flask, followed by the sequential addition of 0.4 g of copper sulfate, 6 g of potassium sulfate, and 20 mL of concentrated sulfuric acid. The mixture was digested until the solution turned green. After cooling to room temperature, 20 mL of distilled water was added. The solution was then transferred to a 100 mL volumetric flask. The digestion flask was rinsed with a small amount of water, and the rinsing solution was also added to the volumetric flask, which was then filled to the mark with distilled water.

For distillation, 1 g/L methyl red ethanol solution and hydrochloric acid were added to the distillation unit and heated to boiling. In the receiving flask, 10 mL of boric acid solution and one drop of mixed indicator were added, ensuring the lower end of the condenser tube was immersed in the solution.

Precisely 3 mL of the digested sample solution was introduced into the reaction chamber. A small beaker was rinsed with distilled water, and the rinsing solution was also added to the chamber. Then, 10 mL of sodium hydroxide solution was added to initiate distillation. After 10 min of distillation, the receiving flask was lowered to remove the condenser tip from the liquid surface, and distillation was continued for another minute. The condenser tip was then rinsed with a small amount of water. The distillate was immediately titrated with standard hydrochloric acid solution until the color changed to gray-blue. A blank determination was conducted in parallel. The protein content (%) was calculated using Equation (3):

(3) $$X=\frac{(V_{1}-V_{2})\times C\times 0.014}{m\times V_{3}/V_{4}}\times F\times 100$$

X is the protein content (%), V_1_ is the volume of hydrochloric acid used for titration of the sample (mL), V_2_ is the volume of hydrochloric acid used in the blank titration (mL), V_3_ is the volume of digested sample solution taken for analysis (mL), V_4_ is the total volume of the digested solution after dilution (mL), m is the mass of the sample (g), C is the concentration of the standard hydrochloric acid solution (mol/L), F is the nitrogen-to-protein conversion factor (typically 6.25 for general protein estimation), 0.014 is the equivalent mass of nitrogen (g/mol).

### 2.6. Determination of Crude Fiber Content

A 25 g sample of ginger powder was weighed and placed into a 500 mL Erlenmeyer flask. Then, 200 mL of boiling 1.25% sulfuric acid solution was added. The mixture was maintained at a boil for 30 min, with stirring every 5 min. After boiling, the mixture was filtered through linen cloth. The residue was subsequently washed with hot 1.25% sulfuric acid, hot distilled water, and finally with 96% ethanol to remove impurities.

The retained sample along with the filter paper was transferred into an aluminum crucible and dried in an oven at 105 °C. After drying, the sample was cooled in a desiccator and repeatedly weighed until a constant weight was achieved. The crude fiber content was calculated using Equation (4):

(4) $$\mathrm{CFC}=\left(W_{i}-W_{a}\right)\times 100\%$$

CFC is the crude fiber content of the sample (%), W_i_ is the initial dry weight of the sample and filter paper after drying (g), W_a_ is the weight of the residual ash after incineration (g).

### 2.7. Determination of Volatile Oil Content

A total of 500 mL distilled water was added into a round-bottom flask, followed by injection of 1 mL xylene via the side arm of the apparatus. The moisture trap was half-filled with distilled water, and blank distillation was carried out for 30 min at a constant flow rate of 2.5 mL/min. The three-way valve was adjusted to align the upper meniscus of xylene with the zero graduation mark of the receiving tube. After a 10 min cooling period, the initial volume of xylene (V_0_, mL) was recorded.

Subsequently, 30 g ginger powder sample was weighed and loaded into a new identical round-bottom flask. The condenser was reconnected, and steam distillation was performed continuously for 4 h with the distillation rate maintained at 2.5 mL/min. After cooling for 10 min, the volume of the organic phase containing volatile oil (V_1_, mL) was read and recorded. The volatile oil content was calculated using Equation (5):

(5) $$X=100\times \frac{V_{1}-V_{0}}{m}\times \frac{100}{100-\omega }$$

where X = volatile oil content (%), V_0_ = initial volume of xylene added (mL), V_1_ = final volume of the organic layer after distillation (mL), m is the mass of the ginger powder sample (g), ω= moisture content.

### 2.8. Determination of Gingerol Content

Gingerol was extracted and quantified as follows: An exact quantity of 1.0 g of ginger powder was added to 50 mL of methanol and subjected to reflux extraction at elevated temperature for 30 min to extract gingerol compounds. The extract was then centrifuged at 3600 r/min for 15 min, and the supernatant was collected and filtered through a 0.45 μm microporous membrane. Subsequently, the gingerol content was analyzed using HPLC.

The chromatographic conditions were as follows: mobile phase A was acetonitrile, and mobile phase B was 0.1% formic acid aqueous solution. The flow rate was set at 0.5 mL/min, and detection was carried out at 282 nm. The detailed elution conditions are presented in Table 1.

### 2.9. Determination of Antioxidant Capacity

2,2-Diphenyl-1-picrylhydrazyl (DPPH) Radical Scavenging Assay: In a 5 mL centrifuge tube, 1.5 mL of 0.1 mmol/L DPPH methanol solution was introduced. Ginger powder solution and ascorbic acid (VC) positive control were prepared across a concentration series of 0.05–0.50 mg/mL, and 0.3 mL of each prepared solution was carefully mixed with the DPPH methanol solution.

The mixture was incubated in the dark for 30 min, and its absorbance was measured at 510 nm. The radical scavenging activity was calculated using Equation (6).

(6) $$Free\;Radical\;Scavenging\;Activity\left(\%\right)=\frac{A_{0}-A_{1}}{A_{0}}\times 100$$

A_0_ is the absorbance of the blank control (without sample), A_1_ is the absorbance of the sample solution.

2,2′-Azino-bis (3-ethylbenzothiazoline-6-sulfonic acid) diammonium salt (ABTS) Radical Scavenging Assay: In a 5 mL centrifuge tube, 1.8 mL of the ABTS working solution was carefully added. Ginger powder solution and ascorbic acid (VC) positive control were prepared at a concentration series of 0.02–0.20 mg/mL, and 0.1 mL of each prepared solution was added to the working solution and mixed thoroughly. The absorbance of each mixture was accurately measured at 734 nm in the dark. To ensure accuracy and reliability of results, each sample underwent testing in triplicate.

Ferric Reducing Antioxidant Power (FRAP) Assay: In a 5 mL centrifuge tube, 0.5 mL of PBS buffer and 0.5 mL of 1% potassium ferrocyanide solution were added, followed by 0.5 mL each of ginger powder and ascorbic acid (VC) positive-control solutions, which were prepared at a concentration series of 0.05–0.50 mg/mL. The reaction was conducted in a dry thermostat at 50 °C for 20 min. After cooling to room temperature, 0.5 mL of 10% trichloroacetic acid solution was added, and the mixture was centrifuged at 3600 r/min for 15 min. The supernatant was collected, and 0.5 mL of distilled water and 0.1 mL of 0.1% ferric chloride solution were added sequentially. The mixture was vortexed and reacted for 10 min, after which the absorbance was measured at 700 nm. Each sample was tested in triplicate.

### 2.10. Preparation of Standard Samples

The standard sample of Xiaolin Huangjiang powder was prepared using the two-stage drying process (high-temperature rapid drying at 120 °C for 90 min followed by low-temperature thorough drying at 80 °C for 150 min). The obtained dry powder was pulverized and sieved through a 100-mesh screen. Exactly 1 g of ginger powder (accurate to 0.001 g) was weighed using an electronic balance, transferred into a 10 mL brown sample vial, and tightly capped. Each vial was placed into an aluminum foil vacuum bag for vacuum sealing. A total of 300 aliquots were prepared and stored at 4 °C.

Homogeneity Assessment

The homogeneity of the ginger powder was evaluated following the guidelines provided in CNS GB/T 15000.3-2023 [16]. Fifteen packaging units were randomly selected from the beginning, middle, and end of the packaging process. Three subsamples were taken from each unit and analyzed by HPLC following the gingerol extraction method described in Section 2.8. Analysis of variance (ANOVA) was applied for statistical evaluation; the sample was considered homogeneous only when the calculated F-value was below the critical value.

Short-Term Stability Study

Short-term stability was assessed on Days 0, 1, 3, 5, and 7 under 4 °C storage conditions. At each time point, three samples were randomly drawn and each was analyzed in triplicate. Data were analyzed using linear regression analysis; stability was confirmed when the calculated t value was below the critical value.

Long-Term Stability Study

Long-term stability was assessed following the principles of CNS GB/T 15000.3-2023 [16] to examine the stability of characteristic values over extended storage. Samples were stored at 4 °C and analyzed at 0, 1, 2, 3, and 6 months. At each time point, three samples were randomly selected and measured in triplicate by HPLC (conditions as in Section 2.8). Stability was evaluated by linear regression analysis; the sample was considered stable when the calculated t value was below the critical value t and the *p*-value was greater than 0.05.

Characterization (Value Assignment)

HPLC was employed as the analytical method, and a collaborative study was conducted involving eight laboratories (designated as A–H). Each laboratory received three sample vials, and each sample was analyzed in triplicate; the mean value was taken as the final result. The Dixon and Cochran tests were applied to examine the submitted data for outlier and variance homogeneity, respectively. Participating laboratories included China Jiliang University (A), Zhejiang Sci-Tech University (B), Zhejiang University (C), Hangzhou Normal University (D), Zhejiang Fangyuan Testing Center (E), Zhejiang Academy of Inspection and Quarantine Science and Technology (F), Zhejiang Academy of Agricultural Sciences (G), and Zhejiang Fangce Technology Co., Ltd. (Hangzhou, China) (H).

Uncertainty Evaluation

The uncertainty in the preparation of the Xiaolin Huangjiang powder quality control sample comprised contributions from characterization, homogeneity, and stability. The combined standard uncertainty was calculated according to Equation (7)

(7) $$u_{crm}=\sqrt{u_{char,rel}^{2}+u_{sts,rel}^{2}+u_{lts,rel}^{2}+u_{bb,rel}^{2}}$$

### 2.11. Statistical Analysis

Each cultivar–treatment combination was independently replicated three times (i.e., three separate batches of raw ginger procurement, drying, and powder preparation). For each independent replicate, all analytical measurements were performed in triplicate as technical replicates, and the mean value of the three technical replicates was used as the representative value for that independent replicate. Statistical analyses were conducted using the independent replicates as experimental units (*n* = 3 per cultivar–treatment combination), while technical replicates were used solely to estimate analytical variability. Technical replicates were not treated as independent observations in any statistical test.

Each sample was analyzed in triplicate as technical replicates. Data are presented as mean ± standard deviation (SD). A one-way analysis of variance (ANOVA) was performed to evaluate the differences in physicochemical properties among different samples. Duncan’s multiple range test and t-test were used to compare the mean values of various indicators, including protein content, crude fiber, moisture levels, ash content, essential oil concentration, gingerol content, and antioxidant activity. Statistical significance was set at *p* < 0.05 (*) and *p* < 0.01 (**).

All data were analyzed using IBM SPSS Statistics for Windows 27.0 (IBM, Armonk, NY, USA). The homogeneity of quality control (QC) samples was evaluated using the F-test. Linear regression analysis was conducted in Origin 2024 to assess the temporal stability of QC samples. Dixon’s test and Cochran’s test were performed in Microsoft Excel to examine the consistency of QC sample measurements. All figures in this study were generated using Origin 2024 and Microsoft PowerPoint.

## 3. Results and Discussion

### 3.1. Basic Physicochemical Properties of Ginger Powder

#### 3.1.1. Moisture Content of Ginger Powder

**Figure 2:**
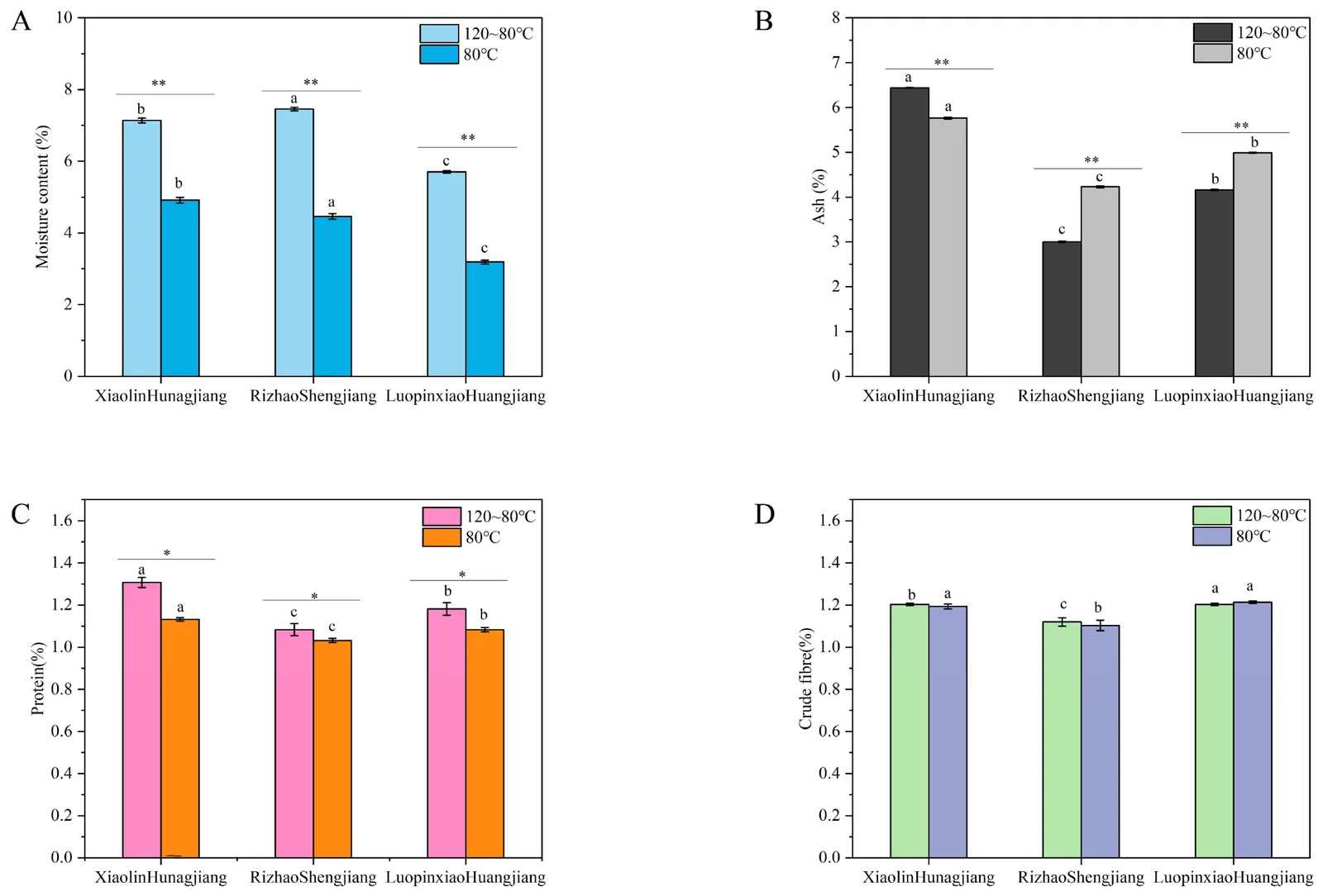
Basic Physicochemical Properties of Ginger Powder. (**A**): Moisture content analysis of ginger powder; (**B**): Ash content determination of ginger powder; (**C**): Protein content evaluation of ginger powder; (**D**): Crude fiber content assessment of ginger powder. Different lowercase letters (a, b, c) indicate significant differences among treatments (*p* < 0.05). Asterisks (*, **) indicate significant differences compared with the control (* *p* < 0.05, ** *p* < 0.01).

Moisture content directly affects the organoleptic properties and quality of ginger powder. An appropriate moisture level helps maintain the integrity of ginger powder cells, which in turn aids in the preservation of gingerol content [17,18]. Moreover, moisture content also influences the aromatic profile of ginger powder—within a certain concentration range, moisture content is positively correlated with the intensity of aroma [17]. As shown in Figure 2A, when the processing temperature was 80 °C, the moisture content of Xiaolin Huangjiang, Rizhao Shengjiang, and Luoping Xiao Huangjiang was 6.58%, 7.15%, and 5.35%, respectively. Under the 120–80 °C combined drying process, the moisture content was 7.14%, 7.46%, and 5.70%, respectively.

#### 3.1.2. Ash Content in Ginger Powder

Ash content reflects the mineral composition of ginger powder and serves as a key quality parameter. Both excessively high and low ash levels can negatively impact quality. High ash content may increase the risk of moisture absorption and spoilage, while low ash content may fail to meet nutritional requirements. Generally, the total ash content in food remains relatively stable, and for ginger powder, it should not exceed 8% [19]. As shown in Figure 2B, when processed at 80 °C, the ash contents of Xiaolin Huangjiang, Rizhao Shengjiang, and Luoping Xiao Huangjiang were 5.76%, 4.23%, and 5.00%, respectively. Under the 120–80 °C process, the ash contents were 6.44%, 3.00%, and 4.16%, respectively. This variation may result from two main factors: (1) moisture reduction may concentrate ash content; (2) differences in mineral profiles among ginger varieties may result in differing responses to high-temperature treatment.

#### 3.1.3. Protein Content in Ginger Powder

Protein content is presented in Figure 2C. At 80 °C, the protein contents of the three ginger powders were 1.13%, 1.03%, and 1.0 8%, respectively. Under the 120–80 °C process, the protein contents increased slightly to 1.31%, 1.08%, and 1.18%. Among the samples, Xiaolin Huangjiang exhibited the highest protein content under the 120–80 °C condition. At 80 °C, Rizhao Shengjiang showed relatively lower protein levels, while the other two maintained values around 1.05%. Overall, the trend in protein content mirrored that of moisture content-protein decreased with low-temperature drying. These findings align with previous studies on thermal treatment of protein-containing food materials, where heat exposure has been reported to cause protein degradation and nitrogen loss [20].

#### 3.1.4. Crude Fiber Content in Ginger Powder

As shown in Figure 2D, when processed at 80 °C, the crude fiber contents of the three ginger powders were 1.20%, 1.10%, and 1.21%, respectively. Under the 120–80 °C process, the corresponding values were 1.20%, 1.12%, and 1.20%. There was no statistically significant difference (*p* > 0.05) in crude fiber content across different processing methods. According to Akshitha et al. [21], the crude fiber content in most commercial dried ginger products ranges between 1.5% and 6.0%. Gloria [22] reported that drying processes can cause a reduction in crude fiber content. In this study, all three ginger powders had crude fiber contents below 1.5%, which would not affect edibility. This is because the primary role of crude fiber in the digestive tract is to support gastrointestinal motility rather than provide nutrition.

### 3.2. Nutritional Composition Analysis of Ginger Powder

#### 3.2.1. Gingerol Content in Ginger Powder

**Figure 3:**
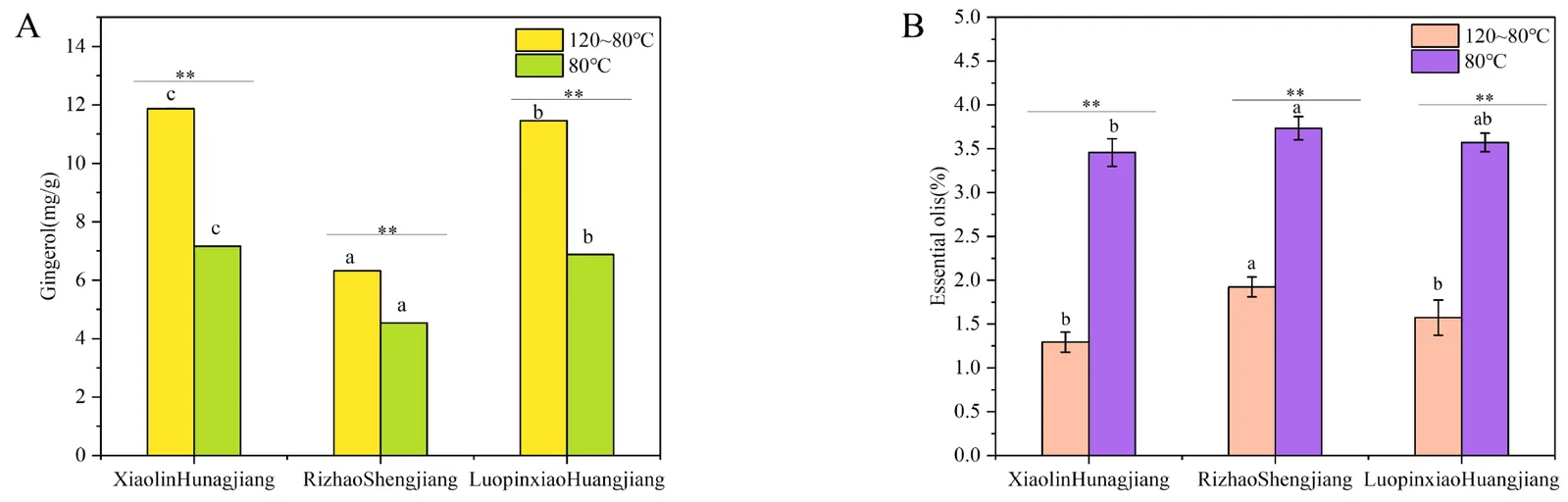
Nutritional composition analysis of Ginger Powder. (**A**): Gingerol content quantification in ginger powder; (**B**): Essential oil content determination of ginger powder. Different lowercase letters (a, b, c) indicate significant differences among treatments (*p* < 0.05). Asterisks (**) indicate significant differences compared with the control (** *p* < 0.01).

As shown in Figure 3A, the gingerol content in all three types of ginger powder decreased under 80 °C drying: Xiaolin Huangjiang, Rizhao Shengjiang, and Luoping Xiao Huangjiang contained 7.12 mg/g, 4.54 mg/g, and 6.89 mg/g of gingerol, respectively. Under the 120–80 °C drying condition, the gingerol content increased to 11.87 mg/g, 11.46 mg/g, and 6.32 mg/g, respectively. The quality of ginger powder is highly sensitive to processing temperature, which primarily affects the gingerol content [23]. Gingerol is a key bioactive compound in ginger, known for its anti-inflammatory [24], antioxidant [25], and anticancer [26] properties. Recent studies also suggest that gingerol may play a role in weight management [26]. Due to its bioactivity, gingerol is prone to degradation during processing and environmental exposure, making its quantification essential for evaluating product quality and functional value.

This variation can be explained by the conversion of phenolic compounds to enol compounds during extended heat exposure, which reduces gingerol content. However, under the 120–80 °C condition, gingerol was better preserved. By modifying the heating temperature and time, the gingerol content in Xiaolin Huangjiang powder increased by 4.7 mg/g. Kubra and Jagan Mohan Rao [27] reported that high temperatures can cause cellular component degradation, thereby releasing more phenolic substances. Other studies have also found that shortening the processing time can enhance gingerol recovery [25]. These findings may explain the improved gingerol yields under the 120–80 °C condition. In contrast, the gingerol degradation at 80 °C may be due to the thermochemical sensitivity of phenolic compounds, which can convert to enol derivatives under prolonged heating [28], leading to a reduction in gingerol levels in the final product.

#### 3.2.2. Volatile Oil Content in Ginger Powder

The main components of volatile oil in ginger include terpenes, flavonoids, and sterols. Studies have shown that ginger volatile oil exhibits strong antibacterial properties and also possesses anticancer potential [29]. As illustrated in Figure 3B, ginger powders processed at 120–80 °C had significantly lower volatile oil contents. When processed at 80 °C, the volatile oil contents of Xiaolin Huangjiang, Rizhao Shengjiang, and Luoping Xiao Huangjiang were 3.46%, 3.74%, and 3.57%, respectively. Under the 120–80 °C drying process, the corresponding values were 1.29%, 1.92%, and 1.57%.

According to the findings of Sellami et al. [30], volatile compounds in plant samples tend to evaporate alongside water during the drying process. The results observed at 80 °C in this study are consistent with their conclusion. However, under the 120–80 °C condition, although the Xiaolin Huangjiang powder had a higher moisture content than Luoping Xiao Huangjiang, its volatile oil content was lower, which may be attributed to rapid volatilization during the initial 120 °C drying phase.

The data clearly indicate that the volatile oil content in ginger powder processed at 80 °C was significantly higher than that of the powder processed at 120–80 °C (*p* < 0.01). Previous studies have suggested that drying temperatures above 70 °C may lead to starch gelatinization in ginger, which affects the retention of volatile oil [31]. Based on these observations, further research is warranted to explore the impact of high-temperature rapid drying followed by low-temperature thorough drying on the preservation of volatile oil.

Combined with the above physicochemical properties, gingerol and volatile oil data of three ginger varieties, Xiaolin Huangjiang showed the best comprehensive functional quality, especially with the highest gingerol accumulation under the 120–80 °C staged drying process. In this study, gingerol content was designated as the primary criterion for evaluating drying performance, given that gingerols are the major bioactive compounds responsible for the pungent taste and pharmacological activities of ginger. Since gingerol content was highest at 120–80 °C, this condition was regarded as the optimal drying strategy with respect to active-compound preservation. As gingerol is the primary bioactive substance responsible for ginger’s antioxidant activity, only Xiaolin Huangjiang powder was selected for subsequent antioxidant capacity evaluation to deeply clarify the influence of different drying regimens on its antioxidant performance. Furthermore, given its superior functional component retention and representative quality characteristics, Xiaolin Huangjiang was exclusively adopted for the subsequent QC sample development work to establish a stable quality control reference standard suitable for high-quality dried ginger powder products.

It should be noted that the identification of the 120–80 °C two-stage drying as the preferred strategy in this study is context-specific and based on a functionally oriented evaluation criterion. While this treatment significantly enhanced gingerol content and antioxidant capacity—the primary quality markers of interest—it also resulted in substantial volatile oil loss compared to constant-temperature drying at 80 °C (e.g., 3.46% vs. 1.29% for Xiaolin Huangjiang). In contrast, 80 °C constant-temperature drying better preserved volatile oils but led to greater gingerol degradation due to prolonged heat exposure. Therefore, the superiority of the 120–80 °C treatment is limited to the criterion of bioactive component (gingerol) preservation and antioxidant activity, and does not imply an overall advantage across all quality attributes. The selection of a suitable drying strategy should depend on the intended application and target quality profile of the final ginger powder product.

### 3.3. Antioxidant Capacity of Xiaolin Huangjiang Powder

**Figure 4:**
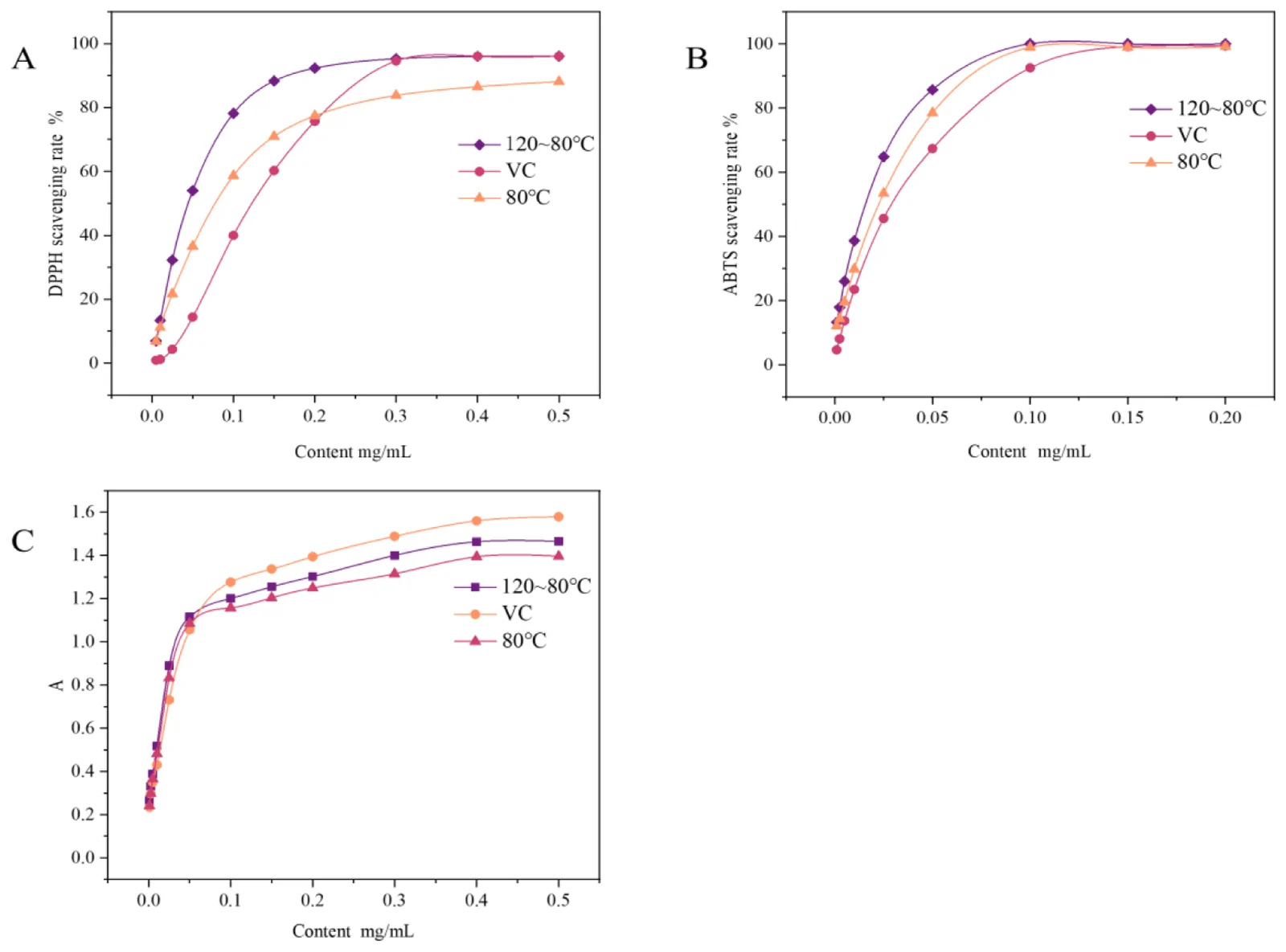
Antioxidant Capacity of Xiaolin Huangjiang Powder. (**A**): DPPH free radical scavenging activity assay; (**B**): ABTS free radical scavenging capacity; (**C**): Ferric ion reducing power assay.

The DPPH and ABTS radical scavenging activities, as well as the ferric ion reducing power (FRAP), of Xiaolin Huangjiang ginger powder were assessed. As shown in Figure 4A, the DPPH radical scavenging rate was positively correlated with ginger powder concentration within the range of 0.001–0.1 mg/mL. At 0.1 mg/mL, the DPPH inhibition rate reached a maximum value of 78.14%. It is evident from Figure 4A that ginger powder prepared at 80 °C exhibited lower antioxidant activity compared to that prepared under 120–80 °C conditions. Figure 4B further demonstrates that the trend in ABTS radical scavenging activity was similar to that observed for DPPH. These results suggest that the antioxidant activity of Xiaolin Huangjiang ginger powder is concentration-dependent, with the 120–80 °C preparation exhibiting significantly higher antioxidant capacity than the 80 °C preparation.

Figure 4C shows the FRAP results for Xiaolin Huangjiang ginger powder within the concentration range of 0.001–0.05 mg/mL. The absorbance was positively correlated with concentration, and within the 0.001–0.01 mg/mL range, the reducing power of ginger powder prepared at both temperature regimes was superior to that of ascorbic acid. However, when the concentration exceeded 0.01 mg/mL, the reducing power of ascorbic acid slightly surpassed that of the ginger powder prepared at 120–80 °C. Additionally, at concentrations above 0.1 mg/mL, the reducing power of the ginger powder prepared at 80 °C was notably lower than that prepared at 120–80 °C. This trend aligns with the DPPH and ABTS results, further confirming that Xiaolin Huangjiang ginger powder possesses significant antioxidant activity, with the highest antioxidant capacity observed in the 120–80 °C preparation.

The experimental results demonstrated that Xiaolin Huangjiang ginger powder exhibits significant antioxidant activity, with the ginger powder prepared using the 120–80 °C drying process showing the strongest antioxidant capacity. Combined with the previous analysis of gingerol content, an association was observed between gingerol levels and antioxidant activity. These findings are consistent with those of Dugasani et al. [32], who also reported a linear relationship between gingerol content and antioxidant potential. Additionally, the antioxidant capacity may also be related to the flavonoid content present in the ginger powder [33].

### 3.4. Quality Control Analysis of Xiaolin Huangjiang Powder

**Figure 5:**
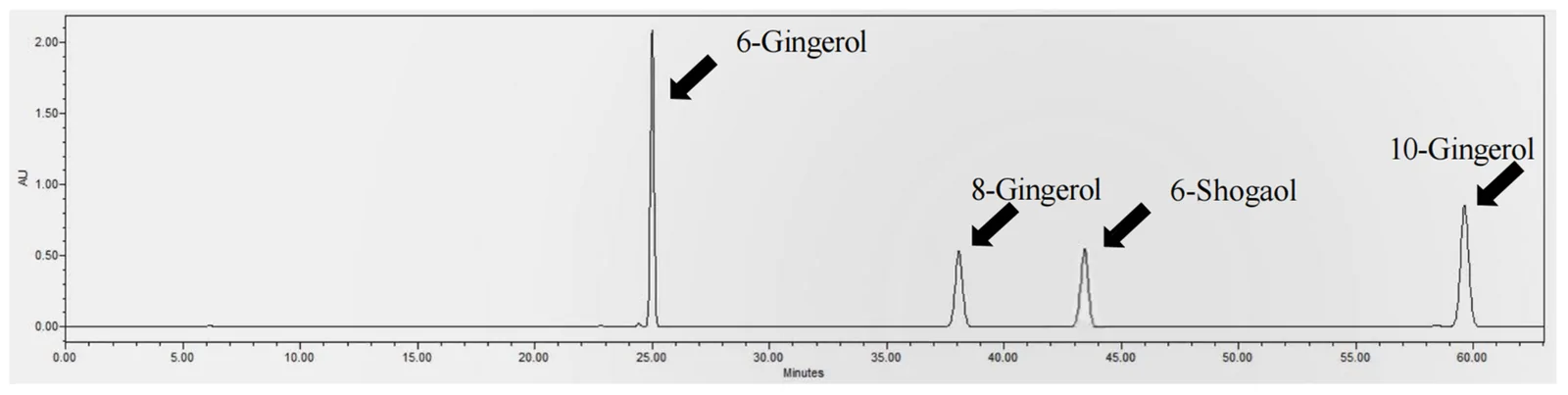
HPLC chromatogram of mixed standard samples containing 6-gingerol, 8-gingerol, 6-shogaol and 10-gingerol.

**Figure 6:**
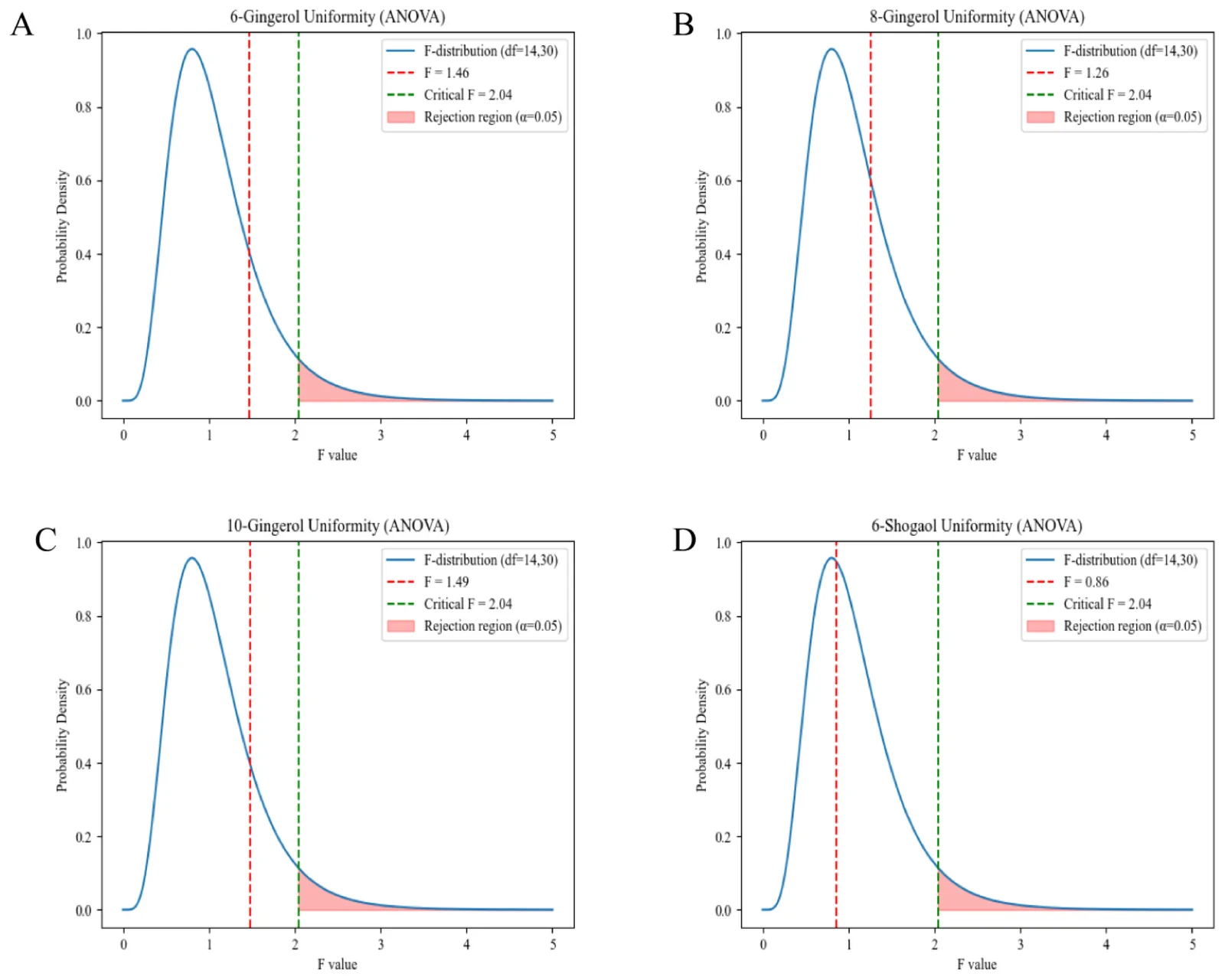
Quality control analysis of Xiaolin Huangjiang powder. (**A**): Homogeneity analysis of 6-gingerol; (**B**): Homogeneity analysis of 8-gingerol; (**C**): Homogeneity analysis of 10-gingerol; (**D**): Homogeneity analysis of 6-shogaol.

In general, gingerols in ginger powder gradually convert into shogaols over time, resulting in the loss of nutritional compounds in the powder [25]. In this study, the homogeneity and stability at 4 °C of Xiaolin Huangjiang ginger powder were evaluated based on its gingerol content. The HPLC chromatogram in Figure 5 achieved complete baseline separation of 6-gingerol, 8-gingerol, 6-shogaol and 10-gingerol mixed standards. As shown in Figure 6, the F-values for the four major components of gingerol were all lower than F_0.05(14,30)_, indicating that the ginger powder prepared using the drying process had good homogeneity, with no significant differences between groups.

**Figure 7:**
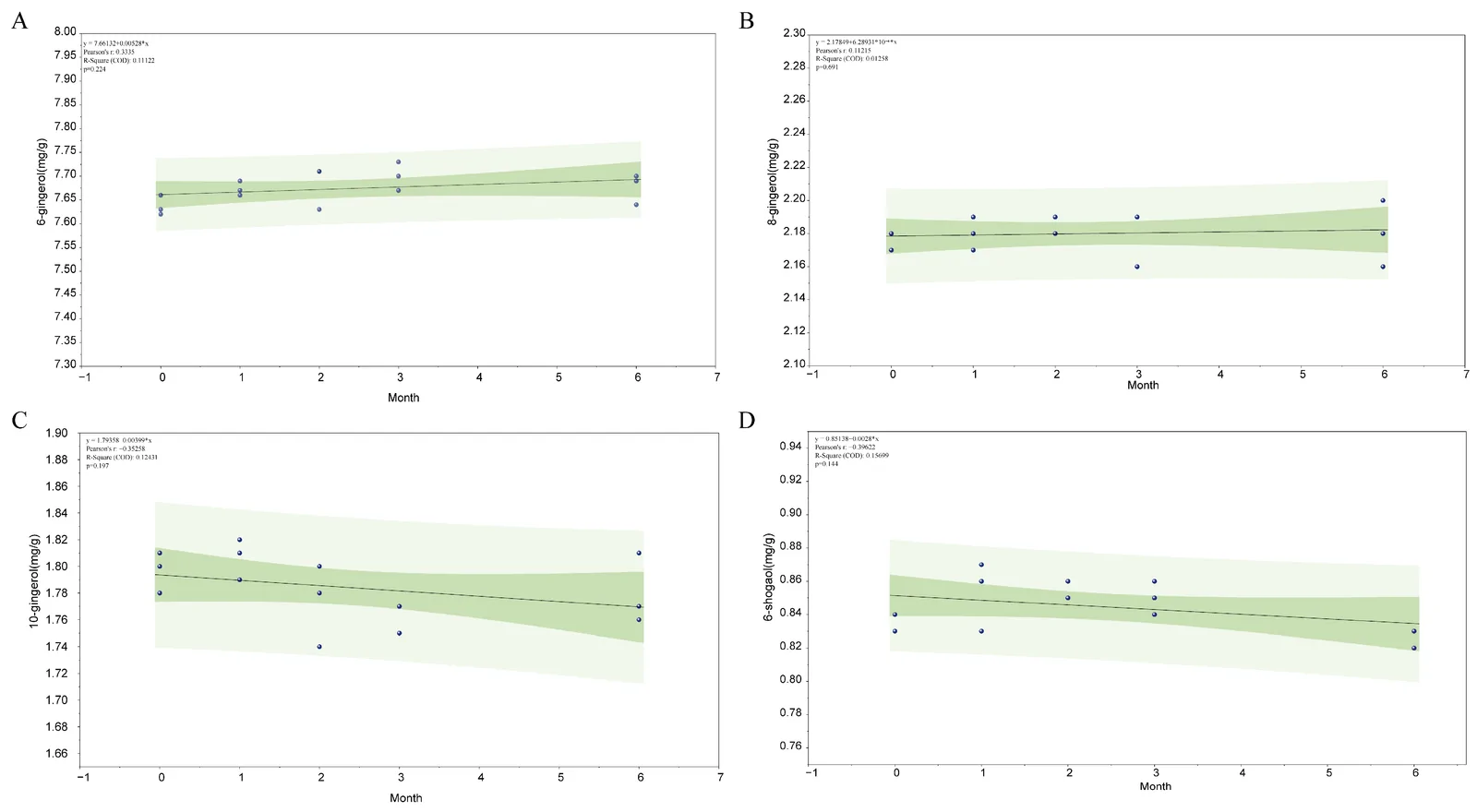
Short-term and long-term stability tests of gingerol content in Xiaolin Huangjiang powder. (**A**): 6-gingerol Long-Term Stability Assessment via Linear Fitting; (**B**): 8-gingerol Long-Term Stability Assessment via Linear Fitting; (**C**):10-gingerol Long-Term Stability Assessment via Linear Fitting; (**D**):6-Shogaol Long-Term Stability Assessment via Linear Fitting.

The results of short-term and long-term stability tests (Figure 7) indicated that the gingerol content in Xiaolin Huangjiang powder showed no significant linear change under 4 °C storage conditions (all t_b1_ values were less than t_0.95,3_). Furthermore, linear regression analysis of the long-term stability data revealed that *p*-values for all major gingerol components were greater than 0.05, suggesting that time had no significant effect on gingerol content. These findings demonstrate that the characteristic compounds in Xiaolin Huangjiang powder, particularly gingerols, remain stable over a six-month storage period. Due to time limitations, the present study was conducted only up to six months; follow-up observations will be continued up to 12 months.

Table 2 shows that in short-term stability, the t_b1_ values of the four components are 1.23, 0.82, 0.75, and 1.06, all within the allowable range, indicating that the sample has good stability under short-term observation conditions. In long-term stability, the t_b1_ values of the four components are 1.06, 0.56, 1.06, and 1.03, all meeting the stability evaluation requirements, indicating that the sample maintains quality stability under monthly storage conditions.

The results show the joint value assignment for Xiaolin Huangjiang powder conducted across eight laboratories (Table 3). The assigned values for 6-gingerol, 8-gingerol, 10-gingerol, and 6-shogaol were determined to be 7.52 mg/g, 2.11 mg/g, 1.69 mg/g, and 0.80 mg/g, respectively. Analysis of the joint value assignment results demonstrates that the gingerol content in the Xiaolin Huangjiang powder QC samples exhibits high stability across different laboratory environments, highlighting the reliability and reproducibility of the value assignment process.

The Dixon and Cochran tests were performed on the assigned value data, revealing no outliers. These results indicate that the quality of Xiaolin Huangjiang powder and its related products is well controlled in practical applications.

Following the guidelines of CNB GB/T 15000.3-2023, we evaluated the uncertainty in the preparation of quality control (QC) samples, primarily considering uncertainties introduced by value assignment (*u_char,rel_*), homogeneity assessment (*u_bb,rel_*) and stability testing (*u_sts,rel_*, *u_lts,rel_*). Using Equation 2, the combined uncertainty of Xiaolin Huangjiang powder was determined as follows: *u_crm,rel,_*_6-gingerol_ = 5.91%; *u_crm,rel,_*_8-gingerol_ = 11.70%; *u_crm,rel,_*_10-gingerol_ = 6.31%; *u_crm,rel,_*_6-shogaol_ = 4.16%.

When the expansion factor (K = 2) was applied, the expanded uncertainties of Xiaolin Huangjiang powder QC samples were calculated as follows:

u*_crm_*_,6-gingerol_ = 0.89 mg/g; u*_crm_*_,8-gingerol_ = 0.49 mg/g; u*_crm_*_,10-gingerol_ = 0.21 mg/g; u*_crm_*_,6-shogaol_ = 0.07 mg/g.

The assigned values for the Xiaolin Huangjiang standard sample components were determined as:6-Gingerol = (7.52 ± 0.89) mg/g; 8-Gingerol = (2.11 ± 0.49) mg/g; 10-Gingerol = (1.69 ± 0.21) mg/g; 6-Shogaol = (0.80 ± 0.07) mg/g.

## 4. Conclusions

This study systematically investigated the effects of two representative drying strategies (constant-temperature drying at 80 °C and 120–80 °C staged drying) on the basic physicochemical properties, nutritional components, and characteristic bioactive compounds of three major ginger cultivars. Significant varietal differences in drying tolerance and quality retention were observed among the tested ginger germplasms. The 120–80 °C two-stage drying effectively increased moisture and protein contents and substantially improved gingerol content in ginger powder compared with continuous low-temperature drying. Notably, prolonged heating at 80 °C led to a pronounced reduction in phenolic bioactive components. In contrast, high-temperature short-time pretreatment in staged drying facilitated the release of intracellular phenolic substances and greatly elevated final gingerol content, although it inevitably caused severe loss of volatile oil. Crude fiber contents remained stable regardless of drying regimens, indicating that thermal processing exerted negligible impacts on dietary fiber characteristics of ginger powder.

Comprehensive comparative screening demonstrated that Xiaolin Huangjiang possessed the best overall quality performance among the three cultivars, exhibiting the highest gingerol accumulation and superior processing adaptability under staged drying. Given that gingerol serves as the core substance responsible for ginger’s antioxidant activity, Xiaolin Huangjiang was exclusively selected for further functional evaluation and quality control development. Antioxidant assays verified that the antioxidant capacities (DPPH, ABTS, and FRAP) of Xiaolin Huangjiang powder were concentration-dependent and significantly enhanced after 120–80 °C staged drying, which showed a clear trend consistent with its elevated gingerol level. Nevertheless, it should be emphasized that the 120–80 °C staged drying was identified as the preferable strategy specifically with respect to functional bioactive components, particularly gingerol content and antioxidant capacity; this treatment resulted in higher volatile oil loss compared to constant-temperature drying at 80 °C. For applications prioritizing aromatic quality, 80 °C constant-temperature drying may be more suitable. The selection of an appropriate drying strategy should therefore be guided by the target quality attributes and intended use of the final product.

Furthermore, this study successfully developed and validated a set of quality control (QC) standard samples for dried Xiaolin Huangjiang powder based on four typical gingerol markers (6-gingerol, 8-gingerol, 10-gingerol, and 6-shogaol). Homogeneity detection, short-term and six-month long-term stability tests confirmed excellent batch uniformity and storage stability of the established QC samples. Eight-laboratory joint value assignment verified high reproducibility and environmental adaptability of the characteristic component values. Combined uncertainty evaluation further quantified the reliable tolerance range of each index, establishing clear and standardized reference values for high-quality dried Xiaolin Huangjiang products.

In summary, this study clarifies the cultivar-specific quality responses of ginger to different drying processes and confirms that Xiaolin Huangjiang is an elite raw material for high-value functional ginger powder production. The constructed QC sample system provides a feasible technical standard for quality evaluation and industrial supervision of dried ginger products, offering valuable guidance for precise cultivar selection and drying-parameter adjustment in ginger postharvest processing.

## Figures and Tables

**Table 1:** HPLC Elution Conditions.

Time (min)	Mobile Phase A (%)	Mobile Phase B (%)
0→10	45	55
10→15	45→48	55→52
15→17	48→60	52→40
17→43	60	40
43→45	60→67	40→33
45→48	67→69	33→31
48→58	69→71	31→29

**Table 2:** Homogeneity and Stability Evaluation of Xiaolin Huangjiang Powder QC Samples.

Component	F	Short-Termt_b1_	Long-Termt_b1_
6-gingerol	1.464	1.23	1.06
8-gingerol	1.256	0.82	0.56
10-gingerol	1.485	0.75	1.06
6-Shogaol	0.858	1.06	1.03

**Table 3:** Interlaboratory Value Assignment Results.

Component (mg/g)	A	B	C	D	E	F	G	H	Overall Mean Value
6-Gingerol	7.53	7.53	7.48	7.63	7.73	7.35	7.59	7.3	7.52 ± 0.141
8-Gingerol	2.08	2.13	2.12	2.18	2.15	2.09	2.09	2.07	2.11 ± 0.038
10-Gingerol	1.72	1.63	1.62	1.64	1.64	1.62	1.74	1.88	1.69 ± 0.091
6-Shogaol	0.76	0.81	0.8	0.85	0.88	0.74	0.82	0.72	0.80 ± 0.055

Note: From A to H respectively: China Jiliang University, Zhejiang Sci-Tech University, Zhejiang University, Hangzhou Normal University, Zhejiang Fangyuan Testing Center, Zhejiang Academy of Inspection and Quarantine Science and Technology, Zhejiang Academy of Agricultural Sciences, Zhejiang Fangce Technology Co., Ltd.

## Data Availability

The original contributions presented in this study are included in the article. Further inquiries can be directed to the corresponding authors.
